# Path Loss Measurement of Outdoor Wireless Channel in D-band

**DOI:** 10.3390/s22249734

**Published:** 2022-12-12

**Authors:** Chengzhen Bian, Weiping Li, Mingxu Wang, Xinyi Wang, Yi Wei, Wen Zhou

**Affiliations:** Key Laboratory for Information Science of Electromagnetic Waves, Department of Communication Science and Engineering, Fudan University, Shanghai 200433, China

**Keywords:** D-band, path loss, outdoor channels, channel model

## Abstract

D-band (110–170 GHz) has received much attention in recent years due to its larger bandwidth. However, analyzing the loss characteristics of the wireless channel is very complicated at the millimeter-wave (MMW) band. Research on D-band wireless channels has been focused on indoor short-distance transmissions, with few studies looking at outdoor long-distance wireless channels. In this paper, we provide the design of the D-band outdoor long-distance transmission system, propose the outdoor line-of-sight (LOS) propagation measurements, and study the outdoor D-band propagation loss characteristics with distances up to 800 m. The path loss model uses the Floating Intercept (FI) and the Close-In (CI) model is established based on the least square method. In the CI model, the path loss exponent is greater than 2 and increases with frequency, while in the FI model, the path loss exponent has no apparent frequency dependence. The results show that D-band path loss in long-distance outdoor scenarios is greater than that in free space, indicating that the propagation condition is worse than in free space. The results show that both models have similar performance. Under this basis, the model with the smallest number of parameters would be the optimal choice. In addition, these results prospectively provide a theoretical model for designing and optimizing high frequency mm-wave propagation measurements at a distance of 200 m and beyond.

## 1. Introduction

**Figure 1:**
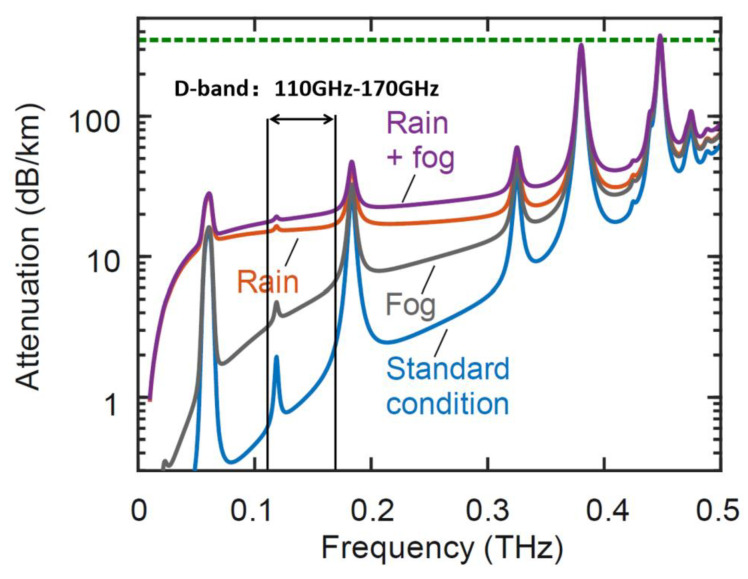
Free-space path atmospheric attenuation.

With the advent of the era of Internet of Everything, more and more things, people, and data are connected via the Internet, which has lead to increasing demand for more authorized wireless spectrum or shared spectrum resource access. The millimeter-wave (MMW) spectrum source is a critical device for a variety of application systems, such as symbiotic sensing and communications, molecular imaging, atmospheric remote sensing and detection, and scaled radar range systems. As a part of MMW resources, the D-band spectrum with its broad bandwidth has become a research hotspot in recent years. Not only do we see a growing attention to D-band as a potential spectrum resource, but there is also a fast-growing number of related channel measurement experiments. D-band radios operating at 110–170 GHz offer benefits, such as high capacities and high antenna gains with a small footprint [1,2,3,4]. The narrower transmit beam makes it more accurate when viewing smaller targets, and also reduces the risk of signal eavesdropping and inter-user interference [5,6,7,8,9,10]. Moreover, it can be seen from Figure 1 that under the standard environment, the atmospheric attenuation does not exceed 2 dB/km in the D-band, which is acceptable for power control and can meet the 5G communication indicators to have an excellent potential in future 5G and 6G communication [11].

However, the poor transmissivity and large propagation attenuation of MMW bring challenges to the research and utilization of MMW. For a better design of outdoor wireless pico-cellular communication systems, the characteristics of propagation channels, especially regarding path loss, need to be modeled and explored in detail. D-band channel characteristics are summarized in Table 1.

As shown in Table 1, New York University has done a lot of work on D-band channel measurements. In 2021, NYU Wireless provided a measurement system with dual-mode switching between the real-time spread spectrum and sliding correlation mode. The researchers measured and examined the reflection and scattering characteristics of wave propagation at 140 GHz. Furthermore, in order to simulate ground-to-satellite and ground-to-unmanned aerial vehicle communications at 140 GHz, they measured rooftop surrogate satellite and backhaul. NYU Wireless also evaluated indoor channels (including offices, conference rooms, classrooms, large hallways, open-plan cubicles, elevators, and a factory building) at 140 GHz using this system in conjunction [12,13,14,15]. In an urban microcell setting, the team also ran directionally resolved outdoor wideband measurement operations at 140 GHz [16]. In 2018, Aalto University reported a microcell directional channel at 140 GHz for a large indoor shopping mall environment, arguing that the spatiotemporal characteristics of the strong path are remarkably consistent between the 28 and 140 GHz channels, and they discovered that the channel’s large-scale parameters are comparable at both frequencies [17]. Georgia Institute of Technology conducted a comprehensive analysis of the physical parameters of the terahertz indoor channel, including LOS path loss, power delay angle profile, temporal and spatial characteristics, and correlation between terahertz multipath characteristics [18]. The University of Southern California (USC) conducted a typical office line-of-sight measurement experiment in the frequency range of 140–220 GHz and estimated the path loss exponent and standard deviation of the shading factor of the CI model using the measurement data [19]. USC has also made some progress recently with longer distances and outdoor settings, but only within 100 m [20]. The University of Ghent designed a VNA-based D-band channel sounder with a 60 GHz bandwidth for characterizing the entire D-band radio channel at distances of up to 5 m. The authors present indoor and outdoor propagation measurements and develop D-band channel models for network performance evaluation [21,22,23]. Shanghai Jiao Tong University (SJTU) developed a 140 GHz VNA-based channel measurement system. They conducted directionally resolved channel measurements in a typical indoor meeting room with Tx/Rx distances ranging from 1.8 to 7.3 m, and in an office room with Tx/Rx distances ranging from 3.75 to 20 m. The team analyzed the temporal and angular distribution of MPCs, as well as the correlation between channel parameters and their distribution [24,25].

Meanwhile, some agencies have measured the channel characteristics of D-band, but to our knowledge, few outdoor long-distance D-band channel characterization based on measurements have been reported [12,13,14,15,16,17,18,19,20,21,22,23,24,25,26,27,28,29]. This paper presents the measurements of the D-band outdoor long-distance channels, explores the applicability of the FI and CI models at long distances in the D-band, and also fits the path loss index and shadow fading. The results show that the path loss of the D-band in outdoor long-distance transmissions is greater than that in free space, indicating that the propagation condition is worse than that in free space. Furthermore, in the CI model, the path loss exponent is greater than 2 and increases with frequency, while in the FI model, the path loss exponent has no apparent frequency dependence. This paper presents prospective and referential outdoor measurement results for D-band channels with large outdoor radio propagation distances up to 800 m.

## 2. Methods

**Figure 2:**
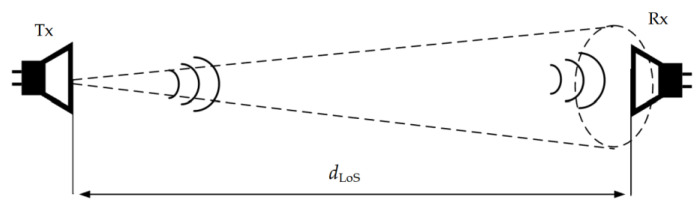
LOS propagation in free space.

The electromagnetic waves propagation in Free-space is shown in Figure 2.

According to the classic Friis transmission equation [30], the relationship between the transmit and the receive power of a wireless link consisting of transmit and receive antennas can be obtained. Its expression is given as:

(1) $$P_{r}=P_{t}+G_{t}+G_{r}-L$$

where, *G_t_* and *G_r_* represent the gains of the transmit and receive antenna respectively; *P_r_* and *P_t_* represent the transmit and receive power; and *L* is free-space path loss (FSPL).

The FSPL model describes the channel propagation characteristics in an ideal propagation environment. Its expression is given as:

(2) $$FSPL(d,f)=20\log_{10}(4\pi df/c)$$

where, *d* is the wireless transmission distance; *f* is the transmission frequency; and *c* is the speed of light.

It can be seen from the above formula that the free space path loss is only related to the transmission distance *d* and the frequency *f*. When the transmission distance or frequency doubles, the loss is increased by 6 dB. The free-space propagation model is suitable for a wireless environment with an isotropic propagation medium (such as a vacuum), and, therefore, is not an ideal model to apply to real scenarios.

Instead, path loss in the real world is highly dependent on the transmission environment. The FSPL model is not accurate enough to describe the actual propagation characteristics. In 5G, two path loss models—the short-range reference (CI) model and the floating intercept (FI) model—are commonly used to characterize the power attenuation during propagation. Both the CI and the FI models are single-frequency path loss models. They can only simulate the path loss at a certain frequency. The 1 m CI model (3) is one of the most frequently used large-scale path loss models to predict the signal strength over distances for various frequencies [31,32]:

(3) $$PL^{CI}(f,d)=FSPL(f,d_{0})+10\beta \log10(\frac{d}{d_{0}})+X_{\sigma }^{CI},d>d_{0}$$

where, *FSPL* is the free space path loss at carrier frequency *f* with *d*_0_ = 1 m; *d*_0_ is a reference distance; β is the path loss exponent that characterizes the dependence of path loss on *d*; and $X_{\sigma }^{CI}$ is the large-scale shadow fading that can be modeled as a zero-mean Gaussian distributed random variable with standard deviation *σ* (in dB).

The CI model has only one variable (β), which can be obtained by the least squares linear fitting method, that is, fitting the measurement data with the smallest error. Therefore, the complexity of the CI model is relatively low.

The FI model is used in the WINNER II and the 3rd Generation Partnership Project (3GPP) [33,34]. Its expression is given as:

(4) $$PL^{FI}(f,d)=\alpha +10\beta \log10(\frac{d}{d_{0}})+X_{\sigma }^{FI},d>d_{0}$$

where, α is a floating intercept in dB that represents the free-space path loss at *d*_0_ = 1 m; *d*_0_ is a reference distance; β is the path loss exponent; and $X_{\sigma }^{FI}$ is the large-scale shadow fading. Path loss model parameters α, β, and *σ* are estimated by the least squares linear fitting method. Similar to the CI model, two variables (α and β) can be obtained by minimizing *σ*. 

## 3. Measurement Setup

**Figure 3:**
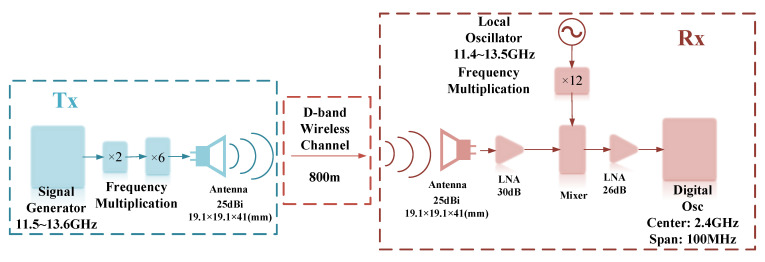
Block diagram of the D-band MMW 800 m transmission experiment system.

The experimental setup of the D-band millimeter-wave transmission system is shown in Figure 3. The signal generator generates an intermediate frequency (IF) signal from 11.5 GHz to 13.6 GHz, which passes through a six-multiplier and a two-multiplier (and is thus multiplied by 12) to reach D-band wavelengths (138 GHz to 163.2 GHz). D-band signals are transmitted to free space via a standard horn antenna. At the Rx-side, another ideal horn antenna that is same as the Tx-side one is used for receiving the signals at the same polarization direction, and the received signal is boosted via a low noise amplifier (LNA) with a gain of 30 dB. A radio frequency (RF) signal at 11.4~13.5 GHz generated by a signal generator is multiplied 12 times to 136.8~162 GHz. Both the intrinsic signal and the received signal are sent to the mixer to down-convert the received signal and generate an IF signal at 1.2 GHz, which is amplified by an electric amplifier with a gain of 26 dB. Finally, its center frequency and received power are observed by an electric spectrometer. The measurement parameters are summarized in Table 2.

This D-band transmission system was implemented in an outdoor LoS environment with clear weather. It was carried out on road on the north side the Guanghua Building in the Handan Campus of Fudan University. Outdoor measurement environments include trees, smooth tiles, concrete floors, metal lampposts, concrete building walls, pedestrians, bare soil ground, concrete pillars, and vehicles. 

**Figure 4:**
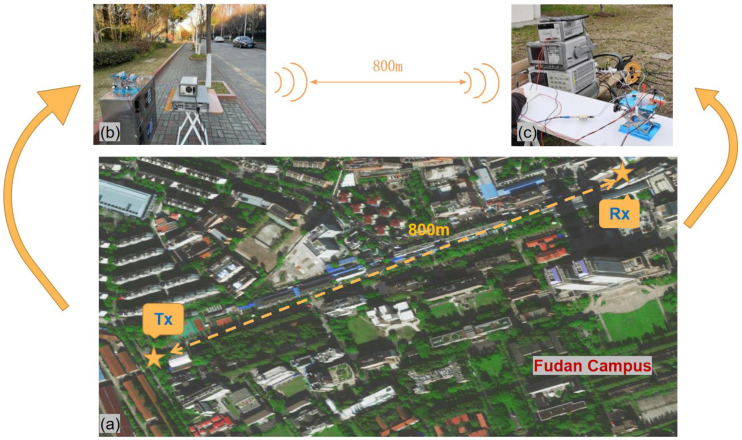
(**a**) Satellite map of D-band transmission 800 m wireless link on Fudan campus; (**b**) photo of Tx-side; (**c**) photo of Rx-side.

As shown in Figure 4, in our measuring experiment, the height of the antennas of the transceiver are set to 1 m. A laser and a telescope pointer were used for calibration to ensure that the antennas on the Tx-side and Rx-side were aligned. The Tx-side moves in a straight line different to set measurement distances at 100 m, 200 m, 400 m, and 800 m, while the Rx-side is fixed. At each measurement location, received power at seven frequency points from 138 GHz to 163.2 GHz were measured. 

## 4. Results

**Figure 5:**
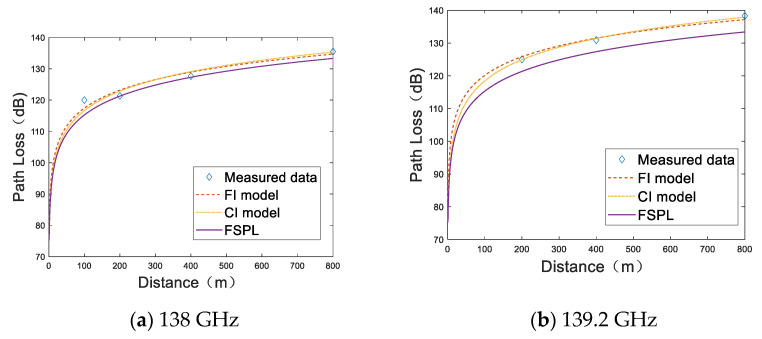

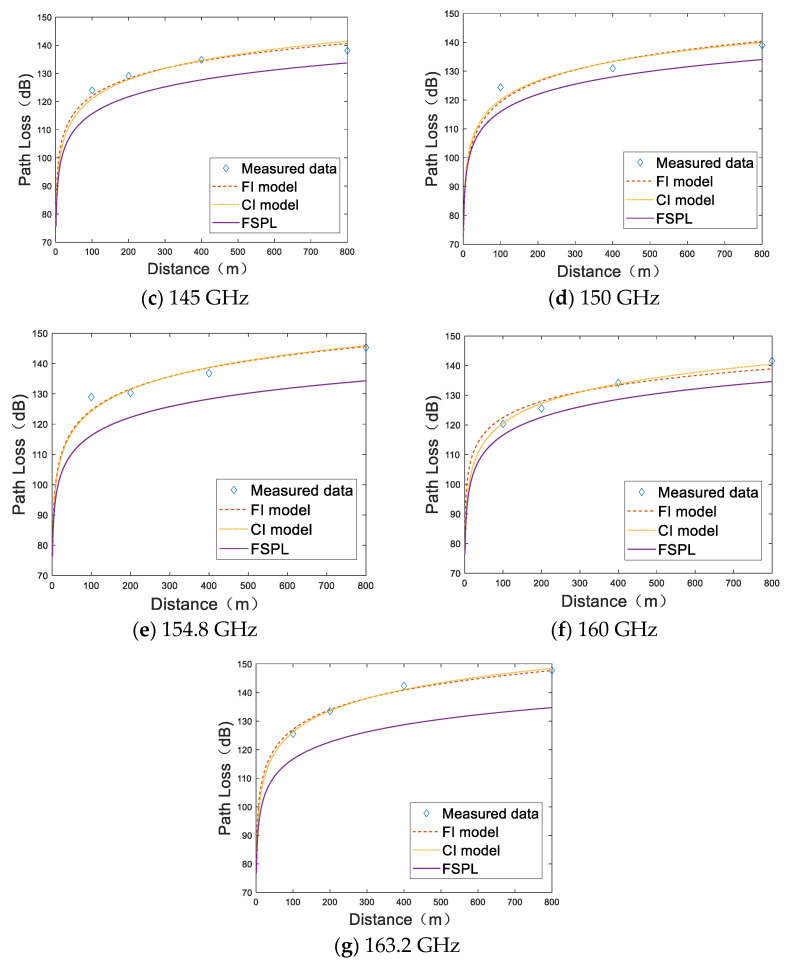
Path loss at 7 frequency points. The diamond represents the path loss sample at a certain distance, and the dotted, dot, and solid lines represent the FI, CI, and FSPL models, respectively. (**a**) 138 GHz; (**b**) 139.2 GHz; (**c**) 145.2 GHz; (**d**) 150 GHz; (**e**) 154.8 GHz; (**f**) 160.8 GHz; (**g**) 163.8 GHz.

**Figure 6:**
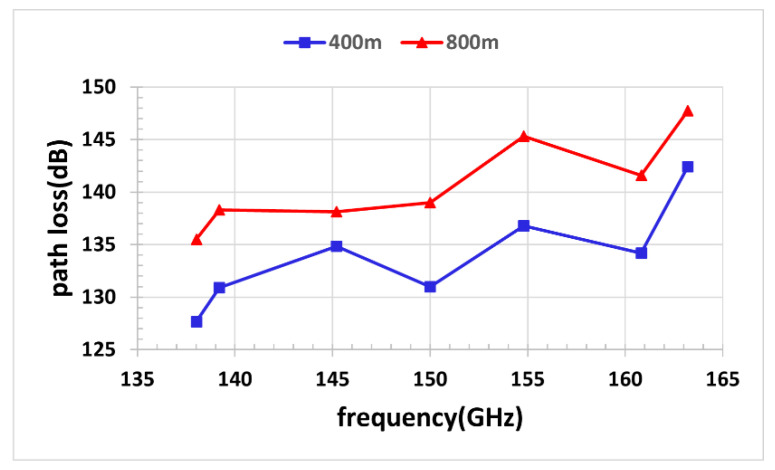
Path loss at distances of 400 m and 800 m.

Figure 5a–g show the path loss samples at 7 different frequency points with a range of 138 GHz~163.2 GHz using the path loss models of FI, CI, and FSPL. To make the figures look clear, 10 measured path loss samples at each distance we taken and averaged, and the averages are presented by the diamonds. It can be observed that the experimentally measured path loss is greater than the theoretical path loss value in free space at all frequency points. The fitting results of the two models are similar and basically coincide at 138 GHz. We can observe that our measurements at 138 GHz agree with FI, CI, and FSPL models. The measured path loss fluctuates more as frequency and distance increase. The measured path loss curves deviate from the free space path loss curves up to 13.1 dB at 163.2 GHz. At the other frequencies, our measurements imply less differences from the FSPL model. Figure 6 shows the path loss at distances of 400 m and 800 m. 

**Figure 7:**
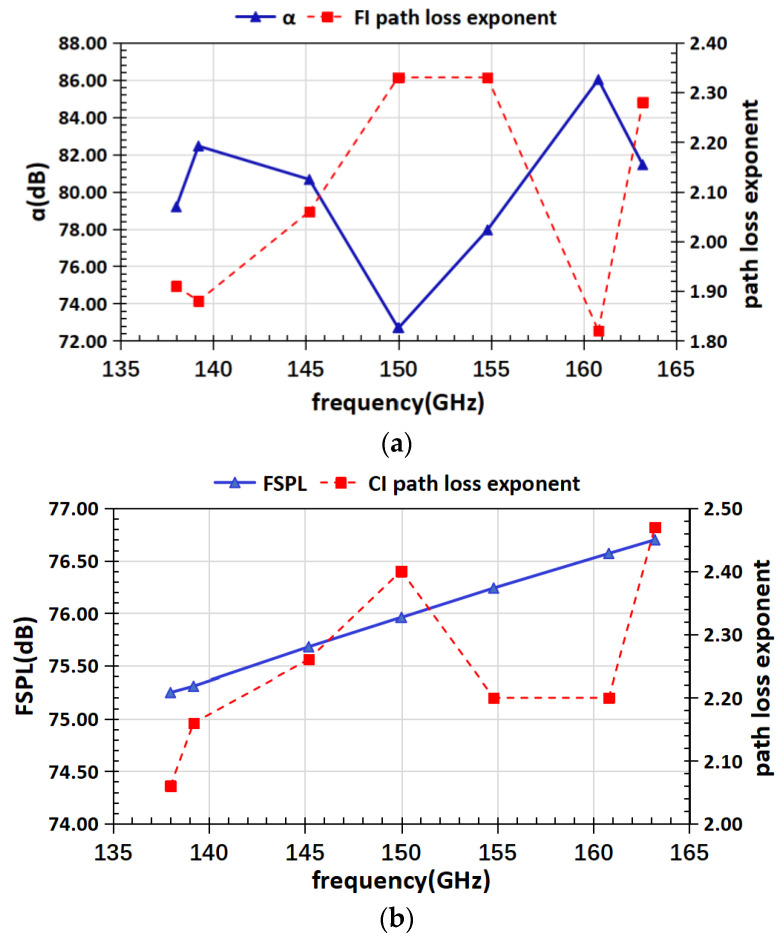
(**a**) FI model path loss model parameters; (**b**) CI model path loss model parameters.

To describe MMW signal path attenuation in D-band as a function of distance under different frequency points in outdoor environments, we estimated the path loss model parameters of the FI and CI models, which are given in Table 3. The CI model has no significant statistical difference compared to FI model, as described in the theoretical analysis. It can be seen that the β parameter of the FI model is around 2 at all frequencies. This differs slightly from the theoretical free space path loss exponent of 2, but its frequency dependence is insignificant. The minimum path loss index is 1.82 at 160.8 GHz, and the maximum path loss index is 2.33 at both 150 GHz and 154.8 GHz. The parameters of the CI model are greater than 2 at all frequency points, the minimum is 2.06 at 138 GHz, and the maximum is 2.47 at 163.2 GHz. Figure 7 demonstrates the variation of the reference distance path loss and path loss exponent at different frequencies in CI and FI models.

Many factors can lead to instability in measurement campaigns. Firstly, the path loss exponent of the test band is extremely sensitive to scatter differences in the measurement environment; specifically, outdoor open environments with ground, trees, and smooth tiles will produce abundant reflections, leading to multipath fading. Furthermore, the strong directionality of millimeter wave beams can also create device alignment problems, especially when the distance is set at 200m and more. The 10° horn antenna with a wide antenna beam used in these measurements also make it harder for antennas to perfectly align. When the distance is greater than a certain value, the signal interferes with ground reflection and direct transmission. The greater the distance, the more reflection paths are generated, and the greater the fluctuation of the received signal.

## 5. Conclusions

This paper presents a D-band MMW transmission measurement campaign in a long-distance outdoor line-of-sight environment with a measurement distance range of 100 m to 800 m. We fit the path loss modeling results at seven frequencies using path loss samples. The performance of the two models is similar in outdoor long-distance scenarios and the path loss exponent for each model differs slightly from the free space path loss exponent. This work provides a reference for future exploration of communications in the D-band frequency range at longer distances. In our future work, we plan to investigate other outdoor scenarios and the propagation characteristics of D-band MMW signals in non-line-of-sight environments.

## Figures and Tables

**Table 1:** Reference Review of Exploring Channel Characteristics.

	Frequency	Antennas	Environment
New York University	142 GHz	27 dBi narrow-beam horn antennas (TX and RX)	1–40 m Indoor offices, conference rooms, classrooms, long hallways, open-plan cubicles, elevators, and the factory building [12,13,14,15]; 25–117 m Outdoor Urban Microcell (UMi) area [16]
Aalto University	140 GHz	19 dBi horn (RX) and 2 dBi bicone (TX) antennas	3–65 m Indoor shopping mall [17]
Georgia Institute of Technology	140 GHz	22–23 dBi horn antennas (TX and RX)	0.3–0.86 m Indoor office [18]
University of Southern California	140–220 GHz	21 dBi narrow-beam horn antennas (TX and RX)	0.5–5.5 m Indoor office [19]; 100 m Urban [20]
Ghent University	110–170 GHz	23 dBi horn antennas (TX and RX)	1–5 m Outdoor [21]; 0.5–8.5 m Indoor office and laboratory [22,23]
SJTU	130–143 GHz	25 dBi horn (RX) and 16 dBi bicone (TX) antennas	1.8–20 m Indoor meeting room and office [24,25]
This work	138–165 GHz	25 dBi narrow-beam horn antennas (TX and RX)	100–800 m Outdoor street

**Table 2:** D-band transmission system specifications.

Specifications	Values						
Center Frequency (GHz)	138	139.2	145.2	150	154.8	160.8	163.2
LO frequency (GHz)	11.5	11.6	12.1	12.5	12.9	13.4	13.6
IF frequency (GHz)	1.2						
Tx/Rx antenna gain (dBi)	25						
Tx/Rx azimuth HPBW	E plane: 9° H plane:10°						
Tx/Rx polarization	Horizontal						
Tx/Rx caliber (mm^2^)	17.5 × 13.6						
Tx/Rx projection diameter (mm)	19.1						
EA1 gain (dB)	30						
EA2 gain (dB)	26						

**Table 3:** Path loss model parameters.

Frequency (GHz)	FI Model			CI Model		
	*α*	*β*	*σ^FI^*	*FSPL* (*f*,*d*_0_)	*β*	*σ^CI^*
138	79.19	1.91	1.75	75.25	2.06	1.98
139.2	82.45	1.88	0.87	75.31	2.16	0.33
145.2	80.66	2.06	1.68	75.68	2.26	2.33
150	72.7	2.33	3.33	75.96	2.40	2.63
154.8	77.95	2.33	2.46	76.24	2.20	2.51
160.8	86.02	1.82	2.13	76.57	2.20	1.02
163.2	81.45	2.28	1.10	76.70	2.47	0.82

## Data Availability

Not applicable.
